# Supplementary material for: Mechanisms governing the pioneering and redistribution capabilities of the non-classical pioneer PU.1
Source: Nat Commun. 2020 Jan 21;11:402. doi: 10.1038/s41467-019-13960-2 (PMC6972792; doi:10.1038/s41467-019-13960-2)
Supplement: Supplementary file 7 — Source data [file 41467_2019_13960_MOESM7_ESM.zip › Source_Data/Figure6/Figure6B_MotifScanOutput/FLI1_PU.1/homerResults/motif23.similar.html]

motif23

## Information for motif23

A
G
C
T
A
G
T
C
C
G
A
T
A
C
G
T
A
G
T
C
A
T
G
C
C
G
A
T
A
T
G
C
A
T
G
C
A
G
C
T
T
G
A
C
A
G
T
C
C
G
A
T
  
Reverse Opposite:  

C
G
T
A
A
C
T
G
A
C
T
G
T
C
G
A
A
T
C
G
T
A
C
G
G
C
T
A
A
T
C
G
T
C
A
G
G
T
C
A
C
G
T
A
C
T
A
G
C
T
G
A
  

|  |  |
| --- | --- |
| p-value: | 1e-55 |
| log p-value: | -1.280e+02 |
| Information Content per bp: | 1.754 |
| Number of Target Sequences with motif | 386.0 |
| Percentage of Target Sequences with motif | 3.88% |
| Number of Background Sequences with motif | 461.1 |
| Percentage of Background Sequences with motif | 1.26% |
| Average Position of motif in Targets | 101.2 +/- 53.0bp |
| Average Position of motif in Background | 117.9 +/- 57.8bp |
| Strand Bias (log2 ratio + to - strand density) | -0.3 |
| Multiplicity (# of sites on avg that occur together) | 1.00 |
| Motif File: | file (matrix) reverse opposite |

### Similar de novo motifs found

|  |  |  |  |  |  |  |  |
| --- | --- | --- | --- | --- | --- | --- | --- |
| Rank | Match Score | Redundant Motif | P-value | log P-value | % of Targets | % of Background | Motif file |
| 1 | 0.757 | C G T A A C T G G C T A C T A G C T A G C G T A G C T A C T A G A C T G C G T A C G T A A C T G C T G A | 1e-43 | -99.576280 | 1.53% | 0.25% | motif file (matrix) |
| 2 | 0.858 | C T G A C A T G T A C G C T G A C A T G T A C G C T G A C A T G T A C G C G T A C A T G T C A G T C G A | 1e-26 | -59.908836 | 1.90% | 0.65% | motif file (matrix) |
